# Effects of different *N*-acyl-serine lactone signaling molecules on the performance of anaerobic granular sludge

**DOI:** 10.1039/d1ra07885b

**Published:** 2022-02-15

**Authors:** Wenhao Dang, Meiling Li, Wencai Fu, Kaili Zhu, Hui Liu, Jinxia Yuan, Wenhua Gao, Guoning Chen, Zhiwei Wang

**Affiliations:** Key Laboratory of Clean Pulp & Papermaking and Pollution Control of Guangxi Province, College of Light Industry and Food Engineering, Guangxi University Nanning 530004 China wangzhiwei@gxu.edu.cn; Guangxi Bossco Environmental Protection Technology Co., Ltd Nanning 530007 China; College of Environmental Science and Forestry, State University of New York Syracuse USA; School of Light Industry and Engineering, South China University of Technology Guangzhou 510006 China

## Abstract

Exogenous addition of acyl-homoserine lactone (AHL) signaling molecules can improve or inhibit the methane production performance of anaerobic granular sludge (AnGS) by quorum sensing (QS). To explore the specific effect of AHLs on AnGS, 2 μM of signal molecules were added to the reactor and we analyzed their effects on AnGS biodiversity, extracellular polymeric substance (EPS), specific methanogenic activity (SMA) and chemical oxygen demand (COD) removal rate of AnGS. The results indicated that the four types of AHLs improve the COD removal rate, SMA and organic composition of AnGS. The addition of *N*-(β-ketocaproyl)-dl-homoserine lactone (3O-C6-HSL) yielded the greatest increase in methanogenic activity, reaching a maximum of 30.83%. The four types of AHLs stimulate the secretion of EPS in AnGS by group sensing regulation. The addition of *N*-hexanoyl-l-homoserine lactone (C6-HSL), *N*-octanoyl-dl-lactone (C8-HSL) and 3O-C6-HSL induced the enrichment of *Actinobacteria*. Thus, the process of hydrolysis and acidification of AnGS is accelerated. The addition of *N*-butyryl-dl-homoserine lactone (C4-HSL), C6-HSL and 3O-C6-HSL promote the potential methanogenic metabolic pathway of AnGS. The addition of all AHLs directly or indirectly enhanced the methane metabolism pathway of sludge and improved the specific methane generation activity of AnGS. These results are expected to provide preliminary research data for enhancing the methane production efficiency of reactors and enriching the biological activity of AnGS.

## Abstract 2

Adding trace signal molecule (AHLs) to the reactor can promote the growth of methanogens and increase the microbial abundance of AnGS. This technology may be applied to enhance methanogenesis of AnGS or restore microbial activity of calcified anaerobic granular sludge.

## Graphical abstract

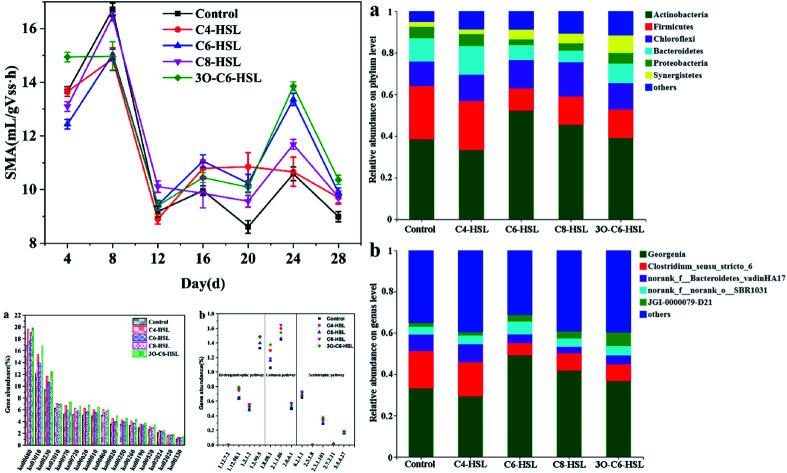

## 1. Introduction

Anaerobic technology plays an important role in wastewater treatment given its advantages, such as wide application range, low energy consumption, high load and small amount of residual sludge.^1^ The upwelling anaerobic sludge bed is a common reactor device. It has the characteristics of low cost and good treatment effect. It is widely used in sewage treatment.^2,3^ However, in the process of treating wastewater from the waste paper recycling industry, the activity of granular sludge is reduced due to the accumulation of Ca^2+^, Mg^2+^ and toxic substances in the wastewater. Problems, such as a decrease in the COD removal rate, weakening of the methane production capacity and calcification of granular sludge, often occur in anaerobic reactors, leading to the failure of effluent to meet the discharge and reuse standards.^4^ Therefore, it is of great significance to improve the activity of anaerobic granular sludge, enhance the efficiency of AnGS treatment and repair the activity of damaged AnGS.

A quorum-sensing system is an important communication tool for microorganisms to communicate with their surroundings.^5^ The microbial population can adjust the cooperative growth and gene expression of the whole biological community through the generation, release and detection of extracellular signal molecules, and quorum-sensing phenomena are noted in both Gram-negative bacteria and Gram-positive bacteria.^6^ Quorum sensing can also occur between Archaea. The study indicates that AHL-based quorum sensing may be used by several species of methanogenic Archaea.^7^ QS is a continuous process. With the increase in bacterial population density, signal molecules also accumulate in the environment. When the content of signal molecules increases to a certain concentration, signal molecules bind to QS receptors, leading to changes in gene expression. This induction of gene expression modulates numerous processes, such as bioluminescence production, biofilm formation, and secondary metabolite production, which can greatly improve the ability of microorganisms to survive in complex environments.^8^ The microbial community of anaerobic granular sludge is a key component of wastewater treatment.^9^ Ding studied the presence and secretion of signal molecules in anaerobic granular sludge under different pH environments, and found that AHLs secretion of anaerobic granular sludge decreased in alkaline environment.^10^ Ma examined sludge in 10 industrial anaerobic reactors and identified four potential universal AHLs in AnGS that are involved in the synthesis of EPS and the granulation of anaerobic sludge.^11^ Lu introduced AHLs into anaerobic reaction and found that it could improve the removal rate of organic matter, but there was a lack of in-depth analysis of the mechanism.^12^

Therefore, four signaling molecules (*N*-hexanoyl-l-homoserine lactone, C6-HSL; *N*-(β-ketocaproyl)-dl-homoserine lactone, 3O-C6-HSL; *N*-butyryl-dl-homoserine lactone, C4-HSL; *N*-octanoyl-dl-lactone, C8-HSL) were added to a sewage reactor to observe their effects on AnGS for continuous culture for one month in this study. The changes of COD removal rate, methanogenic activity and volatile suspended solid/total suspended solid (TSS) of AnGS were compared between the experimental group and control group. The experimental work also quantitatively analyzed the changes of EPS components in sludge, and finally, combined with the changes of microbial community structure, explained the action mechanism of AHLs on anaerobic granular sludge. This study may provide basic data for the development of QS-based strategies to regulate anaerobic particle reactor technology.

## 2. Materials and methods

### 2.1 Anaerobic granular sludge and synthetic wastewater

AnGS used in the experiment was obtained from an upflow anaerobic reactor in a paper mill in Nanning, China. To reduce interference factors in the experiment, sludge with 0.3–0.6 mm particle sizes was screened with 30-mesh and 60-mesh screens, respectively. Four types of AHLS signaling molecules were purchased from Sigma Aldrich Shanghai Trading Company: C6-HSL, 3O-C6-HSL, C4-HSL and C8-HSL. To resuscitate microbes, AnGS was acclimated with glucose as a substrate for one week before the experiment began. The experiment use synthetic wastewater as the reaction substrate, and the composition is as follows: glucose 6.12 g L^−1^, NaHCO_3_ 3.9 g L^−1^, NH_4_Cl 0.72 g L^−1^, KH_2_PO_4_ 0.14 g L^−1^, CaCl_2_ 0.04 g L^−1^, MgSO_4_·7H_2_O 0.045 g L^−1^, FeSO_4_·7H_2_O 0.04 g L^−1^ and pH 7.50 ± 0.20.

### 2.2. Anaerobic digestion experiment

Five anaerobic bottles were used as reactors. A mixture of 150 mL synthetic wastewater and 50 mL sludge was added to each bottle. The bottles are labeled CONTROL, C6-HSL, 3O-C6-HSL, C4-HSL and C8-HSL. The hydraulic retention time (HRT) was 23.5 h. At the end of each cycle, 150 mL of supernatant was replaced with the same amount of fresh substrate, and signal molecules were added. The dosage of each AHL was 2 μM of the total mixture volume (200 mL), and the same amount of deionized water was added to the control group. The bottle was then rinsed with nitrogen for 15 min to drain the air and sealed with a stopper and tape. Three parallel samples were set for each group of experiments. After CO_2_ is removed from the gas generated by the reaction, the volume of methane generated is measured by drainage method. The ambient temperature of the reaction was in a 37 ± 1 °C. COD removal efficiency is measured once at the end of each HRT, and the SMA measurement interval is 4 d. After the experiment, high-throughput sequencing was used to detect AnGS.

### 2.3. Analytical method

#### 2.3.1 VSS, TSS and COD removal rate analysis

The VSS/TSS values were tested with reference to (Zhao *et al.*)'s research.^13^ The COD removal efficiency adopted the research method of Zhou *et al.*^14^

#### 2.3.2 Specific methanogenic activity analysis

The gas produced by the reactor is passed through a saturated sodium hydroxide solution and then collected by an aluminum foil air pocket. Use a syringe to measure the volume of gas.^15^ The SMA value is calculated by cumulative methane production, and then fitted by one-dimensional linear model.^16^ The SMA value was calculated using formula (1):^17^1
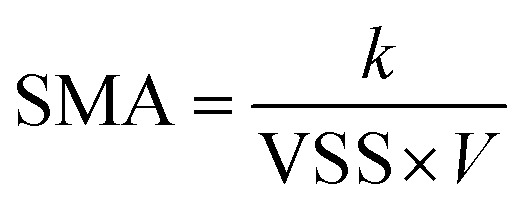
where *k* denotes the slope of the fitted line, mL h^−1^; VSS denotes the average concentration of sludge biomass in the culture bottle, mg L^−1^; and *V* denotes the volume of the mixture, L.

#### 2.3.3 EPS analysis

The extracellular polymers of granular sludge were extracted using the formaldehyde–sodium hydroxide method.^18^ The method was improved as needed. The specific steps were as follows: 2 mL of anaerobic granular sludge was obtained from the reactor, placed into a 20 mL centrifuge tube and washed with distilled water thrice. The supernatant was removed. Then, 12 mL of distilled water and 84 μL formaldehyde were added, and the sludge was vibrated at 200 rpm for 1 h. Then, 280 μL 1 mol L^−1^ NaOH solution was added, and the mixture was vibrated at 200 rpm for 2 h. After centrifugation at 4200 rpm for 15 min, the supernatant was obtained and filtered through a 0.45 μm water system membrane to obtain EPS samples, which were stored for testing. The main components of EPS include polysaccharides, proteins and humus. The polysaccharide content was determined using the anthrone–sulfuric acid method.^19^ To 1 mL of EPS extract, 0.5 mL of anthrone reagent and 5 mL of concentrated sulfuric acid were added, mix evenly, after 10 min in boiling water bath, reaction was cooled, and the absorbance was measured at 625 nm using an UV spectrophotometer (Agilen-8453, Agilent Technologies Inc). The blank group used 1 mL deionized water. The protein and humus contents were determined using the Folin–Lowry method.^20,21^ Beef serum protein and sodium humate were used as standard substances. There are two reagents required for the test, namely reagent A and reagent B. Reagent A as 50 : 1 mixture of solution S1 (2.0 g Na_2_CO_3_ dissolved in 100 mL NaOH solution, concentration 0.1 mol L^−1^) and solution S2 (0.5 g CuSO_4_ dissolved in 100 mL potassium tartrate, concentration 1%) prepared prior to use. Reagent B is Folin–Ciocalteu reagent.

The specific method is as follows: take two test tubes and mark them as 1 and 2. Tube 1 contained 100 μL EPS extract, 900 μL deionized water, and 2 mL reagent A. Tube 2 contained 100 μL EPS extract, 900 μL deionized water, and 2 mL reagent S1. After standing for 10 min, 0.2 mL Folin–Ciocalteu reagent was added to the two test tubes, and heated in a 40 °C water bath for 10 min. The absorbance was measured by UV spectrophotometer at 625 nm after cooling. The blank group used 1 mL deionized water. The absorbance of solution in test tubes 1 and 2 was denoted as *A*_total_ and *A*_blind_ respectively. Calculate the absorbance of protein and humus according to formula (2) and (3). Compared with a standard curve generated with BSA and sodium humate and calculate the protein and humic acid content.

2 *A*_pr_ = 1.25(*A*_total_ − *A*_blind_)

3 *A*_hu_ = *A*_total_ − *A*_pr_

where *A*_total_ denotes the total absorbance; *A*_blind_ denotes the interference absorbance; *A*_pr_ denotes the absorbance of protein; *A*_hu_ denotes the absorbance of humus.

#### 2.3.4 Analysis of the structure of AnGS microorganisms

High-throughput sequencing technology was used to count the microorganisms in AnGS. About 20 g samples were collected before and after the reaction and sent to Majorbio (Shanghai, China) for DNA extraction and amplicon sequencing. The first step was to isolate total genomic DNA from sludge samples using the Qiagen QIAamp Rapid DNA mini kit. In the second step, the primers 341F, 805R, Ar Ba515F and Arch806R of bacteria, archaea and methanogens were used for PCR amplification of extracted DNA. Finally, all PCR products were directly observed on an Agarose gel (2% TAE buffer) and Bandage with the AxyPrep DNA Gel Extraction Kit. Paired-end amplicon library was constructed, and Illumina MiSeq platform was used for sequencing.^22^

Cluster analysis was performed on samples using Uparse software, and the similarity between microorganisms was greater than 97%, which was defined as an OTU (operational taxonomic unit). The Simpson index, Shannon index, the Converge index, Ace index and Chao index of microorganisms were calculated using QIIME software. Statistical analysis of microbial data using R language tools.

The PICRUSt was used to calculate the metabolic function of flora and microbial gene pathway.^23^

## 3. Results and discussion

### 3.1 Effects of AHLs on the performance of AnGS

**Fig. 1:**
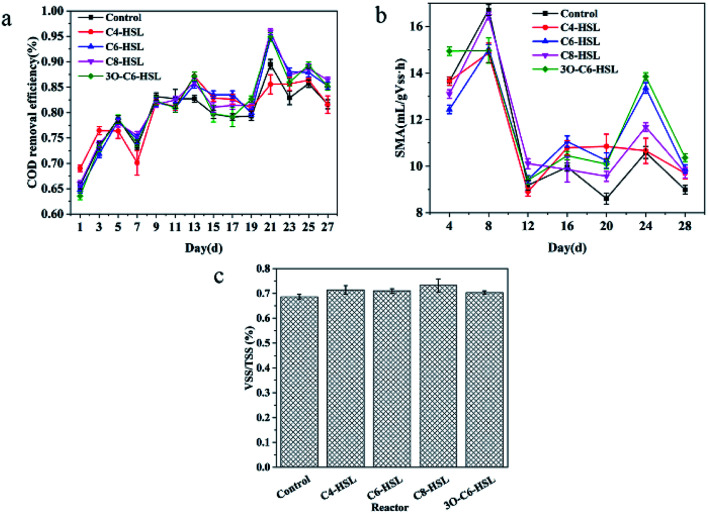
Effects of exogenous acyl homoserine lactones on the functions of AnGS: (a) COD removal efficiency; (b) specific methanogenic activity (SMA); (c) VSS/TSS (volatile suspended solid/total suspended solid).

Fig. 1-a shows the change in the COD removal rate of AnGS after the addition of exogenous signaling molecules in the cultivation process. In the first week of anaerobic reactor operation, there was little difference in COD removal rate between the control group and the four experimental groups. After nine days of operation, the reactor tended to be stable. Compared with the control group, the experimental groups adding C6-HSL, 3O-C6-HSL and C8-HSL all improved the COD removal rate to a certain extent. The COD removal rate of wastewater by granular sludge increases with the progress of cultivation. On day 21, the COD removal rates of the experimental groups with C6-HSL, 3O-C6-HSL and C8- HSL increased by 6.21%, 6.01% and 7.18%, respectively.

Fig. 1-b shows the changes in the specific methane-producing activity of AnGS after the addition of signal molecules in the culture process. After 12 days of culture, the SMA of all the experimental groups with added signal molecules was greater than that of the control group. With the addition of C6-HSL, 3O-C6-HSL, C4-HSL and C8- HSL groups, the SMA increased by 26.06%, 26.18%, 11.10% and 30.83%, respectively. In conclusion, the addition of AHL with different chemical structures improve the COD removal rate and SMA of AnGS on wastewater and play effective roles in promoting the anaerobic reactor. The promotion effects of the four types of AHL differ. Among them, C6-HSL, C8-HSL and 3O-C6-HSL have better COD removal rates, but their effects do not significantly different from each other. C6-HSL and 3O-C6-HSL exhibit better promotion effects than SMA.

Fig. 1-c shows the VSS/TSS of AnGS in different reactors after 28 days of culture. The ratio of VSS/TSS represents the proportion of microbial content in AnGS. In the process of wastewater treatment, it is generally believed that a ratio greater than 0.6 indicates that the sludge has good performance, whereas a ratio less than 0.6 indicates that the sludge has started to calcify.

The VSS/TSS of the experimental groups with the addition C4-HSL, C6-HSL, C8-HSL and 3O-C6-HSL increased by 3.94%, 3.36%, 6.64% and 2.36%, respectively. These results proved that the addition of exogenous signal molecules AHLs can be beneficial to increase the organic components of granular sludge and improve sludge activity. Among them, C8-HSL exhibited the most obvious increase in biological activity.

### 3.2 Effect of AHLs on EPS expression in AnGS

**Fig. 2:**
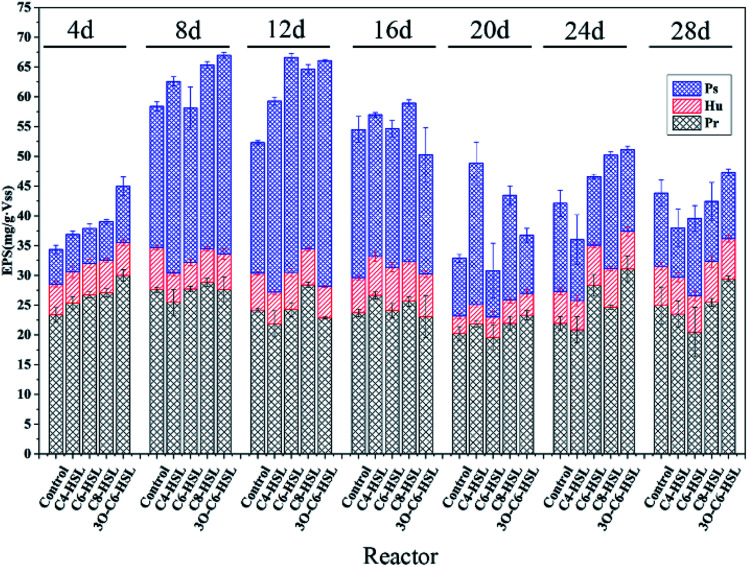
Effects of AHLs on EPS of AnGS.

Fig. 2 shows the effects of different AHLs on EPS expression in AnGS. The presence of extracellularpolymeric substances (EPS), a complex high-molecular-weight mixture of polymers, in pure cultures, activated sludge, granular sludge, and biofilms, has been confirmed and observed using various electron microscopy techniques. EPS is used for self-protection and mutual adhesion, and provides a carbon source and energy for microorganisms in starvation environments.^18^ EPS can also form a buffer layer between the environment and the microbial membrane to protect microorganisms from being damaged by toxic substances to maintain the overall activation of anaerobic granular sludge. The chemical composition of EPS is relatively complex, and the main components assessed in this study include protein, polysaccharide and humic acid. In the Fig. 2, Pr represents protein, Hu represents humic acid, and Ps represents polysaccharide.

As noted in the figure, as the reaction time increases, the overall EPS content exhibited a trend of first increasing and then decreasing. This finding may be related to the activity cycle of microorganisms. Specifically, in the logarithmic growth phase, the content of EPS increases with increasing culture time. After the community structure of anaerobic granular sludge is stable, the content of EPS decreases with increasing culture time. The content of EPS decreases with increasing culture time.^24^

Overall, the addition of exogenous AHLs promoted the secretion of EPS. EPS induces the enrichment of functional microorganisms, increases the extracellular electron transfer active medium (c-Cyts), establishes and speeds up the methane production process of diet, so as to enhance the methane production efficiency of sludge.^25^ AHLs can promote EPS secretion through quorum sensing, thus improving the performance of AnGS.

### 3.3 Effects of AHLs on microbial community structure in AnGS

Table 1 shows the abundance and diversity index of microbial communities in different reactors after 28 days of culture. The coverage index refers to the coverage rate of each sample library. When the coverage index is close to 1, the sequencing results are closer to the actual situation of microorganisms in the sample. The coverage index of this experiment was 0.9961–0.9972. This finding indicates that the actual situation of microbial community structure in the reactor is reflected in the sequencing results.

**Table 1:** Abundance and diversity of microbial communities in different reactors

Reactor	Shannon	Simpson	Ace	Chao	Converge	OTU
Control	3.23	0.170	560.68	566.28	0.9970	449
C4-HSL	2.86	0.240	660.39	640.51	0.9967	436
C6-HSL	4.07	0.056	690.31	688.24	0.9961	574
C8-HSL	4.28	0.034	702.02	703.22	0.9967	596
3O-C6-HSL	3.49	0.106	566.26	557.94	0.9972	478

The Simpson and Shannon indices reflect the diversity of the microbial community in sludge. The greater the Simpson index, the lower the microbial community diversity. The greater the Shannon index, the higher the microbial community diversity.^26,27^Table 1 shows that the addition of C6-HSL, C8-HSL and 3O-C6-HSL is beneficial for improving the microbial diversity of AnGS. C8-HSL exhibited the best promotion effect on AnGS biodiversity. However, the addition of C4-HSL did not improve the microbial diversity of AnGS but decreased it.

Ace and Chao indices reflect the abundance of the microbial community in the sample.^26,27^ The greater the value of these indexes, the greater the abundance of the microbial community. The results in Table 1 show that the abundance of microorganisms in AnGS increased in the experimental group upon the addition of four types of signal molecules, and the increase in C8-HSL was the most obvious.

**Fig. 3:**
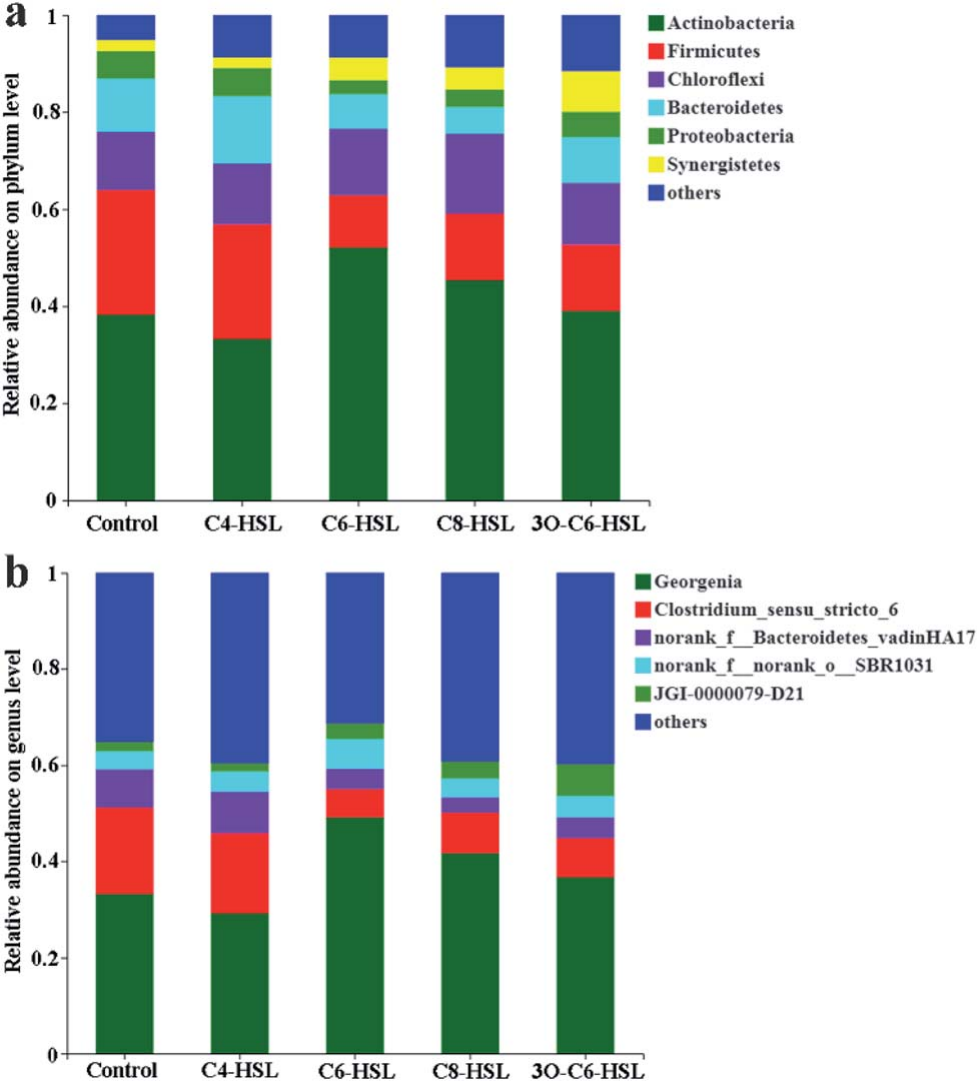
Community analysis of bacteria: (a) analysis of bacterial community structure at the phylum level; (b) analysis of bacterial community structure at the genus level.

Fig. 3 shows the composition analysis of bacteria in AnGS after adding different signal molecules. At the phylum level, *Actinobacteria* was the dominant phylum in all the reactors. However, it should be noted that *Actinobacteria* was slightly reduced in the C4-HSL addition group. Compared with the control group, the addition of C6-HSL and C8-HSL increased *Actinobacteria* by 36.20%, 18.50%. *Actinobacteria* is one of the most common phyla of hydrolytic acidification. The increase in *Actinobacteria* indicates that the addition of C6-HSL and C8-HSL accelerate the hydrolytic acidification process of AnGS.^25^ AHLs is considered a signal molecule for Gram-negative bacteria, but results showed an increase in Gram-positive bacteria. There may be two reasons for this. The chemical structure of AHLs is similar to that of actinomycete signaling molecules, resulting in a stimulant effect.^28^ When Gram-negative bacteria are effected, Gram-positive bacteria have some indirect influence.

58.07%, 46.64% and 46.75% reductions in *Firmicutes* were noted in the C6-HSL, C8-HSL, and 3O-C6-HSL groups, respectively, compared to the control group. In contrast, C4-HSL only yielded a slight reduction. *Synergistetes* can metabolize glucose and acetic acid. In the experimental group with the addition of C6-HSL, 3O-C6-HSL and C8-HSL, the proportion of *Synergistetes* increased significantly. *Synergistetes* can metabolize glucose and acetic acid. This finding indicates that the addition of signal molecules can significantly improve the utilization of glucose and acetic acid and further promote the treatment efficiency of the reactor.^29^ In general, at the phylum level, no significant differences in *Synergistetes* bacteria were noted between the experimental group supplemented with C4-HSL and the control group. However, significant differences in for *Synergistetes* bacteria were noted between the experimental group supplemented with C6-HSL, C8-HSL and 3O-C6-HSL and the control group.

At the genus level, the dominant genus in all the reactors was *Georgenia*. Similarly, the proportion of *Georgenia* microorganisms in the experimental group supplemented with C4-HSL was slightly reduced, whereas the number of *Georgenia* in the other three experimental groups was greater than that in the control group. *Georgenia* increased by 48.18%, 25.63% and 10.52% in the experimental group supplemented with C6-HSL, 3O-C6-HSL and C8-HSL compared with the control group, respectively. *Georgenia* is an actinomycete, which is a hydrolytic acidifying bacterium.^30^ This shows that the addition of C6-HSL, 3O-C6-HSL and C8-HSL can increase the number of actinomycetes and accelerate the hydrolysis and acidification of pollutant substrates in the reactor. Compared with the control group, *Clostridium_Sensu_Stricto_6* in the experimental group decreased by 67.30%, 53.15% and 54.93% with the addition of C6-HSL, 3O-C6-HSL and C8-HSL, respectively. From the data, at the genus level, the microbial community structure of the experimental group added C4-HSL did not change significantly, but *Clostridium_Sensu_Stricto_6* decreased significantly after adding C6-HSL, 3O-C6-HSL and C8-HSL. Combined with the microbial diversity data, it can be seen that the microbial induction effect of C4-HSL on AnGS is not obvious. C4-HSL has no significant effect on the performance of AnGS.

**Fig. 4:**
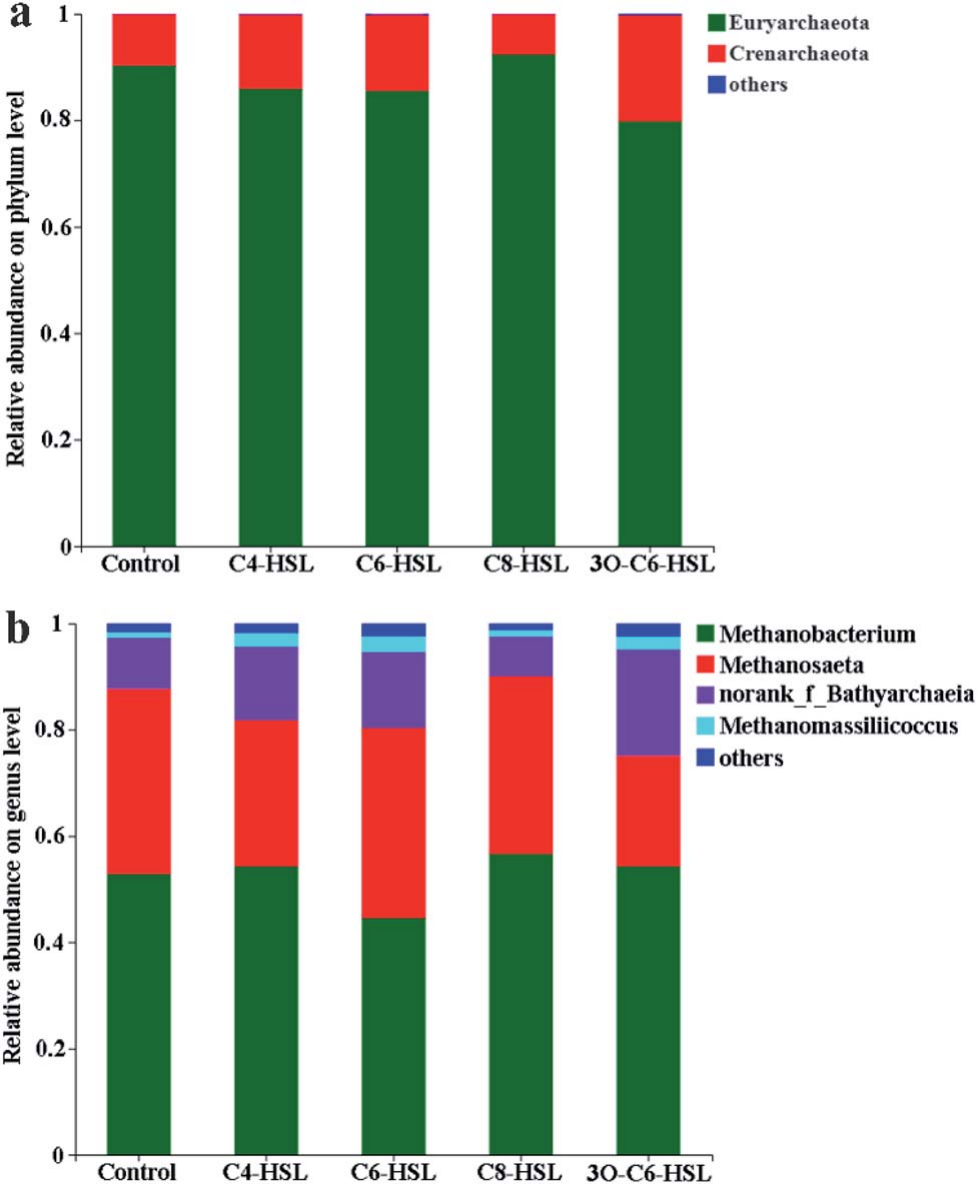
Community analysis of archaea: (a) analysis of archaeal community structure at the phylum level; (b) analysis of archaeal community structure at the genus level.

Fig. 4 shows the composition analysis of archaea in AnGS. The figure demonstrates that the addition of AHL signaling molecules does not affect the main microbial composition of archaea in AnGS, and the dominant phylum remains *Euryarchaeota*. Among the five reactors, *Crenarchaota* (*Archaeota*) accounted for 9.65%, 13.98%, 14.36%, 7.57% and 20.02%, respectively. In the reactors supplemented with C4-HSL, C6-HSL and 3O-C6-HSL, *Euryarchaeota* shows a decreasing trend, whereas *Crenarchaota* shows an increasing trend. At the genus level, the dominant bacterial genera of the five reactors were *Methanobacterium* and *Methanosaeta*.

In the control group and the experimental group supplemented with C4-HSL, C6-HSL, C8-HSL and 3O-C6-HSL, *Methanobacterium* accounted for 52.87%, 54.28%, 44.52%, 56.65% and 54.30%, respectively. *Methanosaeta* accounted for 34.80%, 27.44%, 35.73%, 33.38% and 20.86%, respectively.

In addition to the above two microorganisms, *norank_f_Bathyarchaeia* also accounted for a portion of the reactor, accounting for 9.63%, 13.96%, 14.33%, 7.55% and 19.97% in the five reactors, respectively. *Methanobacterium* is a hydrogenophilic methanogen, *Methanosaeta* is an acidophilic methanogen,^31^ and *norank_f_Bathyarchaeia* is bacterium in the *Bathyarchaeota* genus that may be able to produce methane through a potential methanogenic metabolic pathway (potential methanogenic pathway of *Bathyarchaeota*). However, to date, there has been no evidence that this species metabolizes methane. This information needs to be verified by further physiological and biochemical experiments.^32^

There are competitive quorum-sensing between microbial colonies, which may cause some microbes to be promoted and others to be inhibited.^28^ In the C6-HSL reactor, a 15.79% reduction in *Methanobacterium* was noted compared with the control group. The amount of *Methanosaeta* in the reactor with C4-HSL and 3O-C6-HSL was reduced by 21.15% and 40.06%, respectively, compared with the control group. *Norank_f_Bathyarchaeia* increased by 44.96%, 48.81% and 107.37% in the C4-HSL, C6-HSL and 3O-C6-HSL reactors, respectively, compared with the control group. The results showed that the addition of C4-HSL, C6-HSL and 3O-C6-HSL could increase the content of *norank_f_Bathyarchaeia* in AnGS. Among them, the experimental group supplemented with 3O-C6-HSL exhibited the best growth promotion effect on *norank_f_Bathyarchaeia*. The experimental group supplemented with 3O-C6-HSL exhibited the greatest promotion of methanogenic activity compared with the control group, which may also verify to a certain extent that *norank_f_Bathyarchaeia* metabolizes methane.

### 3.4 Influence of AHLs on methane production pathways in AnGS

**Fig. 5:**
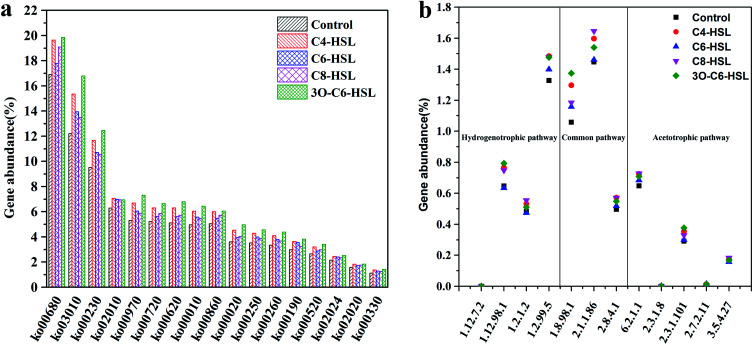
Relative abundance of (a) enriched pathway and (b) genes encoding enzymes involved in methanogenesis.

In this part of the study, the Kyoto Encyclopedia of Genes and Genomes (KEGG) database was used to gain an insight into the methanogenic process. Fig. 5a shows the gene abundance of microbial growth and metabolic pathways in AnGS. Here, the KEGG pathways are: “ko03010”—ribosome, “ko00230”-purine metabolism, “ko02010”-ATP-binding cassette (ABC) transporters, “ko00970”-aminoacyl-tRNA biosynthesis, “ko00620”-pyruvate metabolic, “ko00010”-glycolysis/gluconeogenesis, “ko00860”-porphyrin metabolic, “ko00020”-the citric acid cycle pathway, “ko00250”-the metabolic of alanine, aspartic acid and glutamate. “ko00260”-the metabolic of glycine, serine, and threonine, “ko00190”-oxidative phosphorylation, “ko00520”-amino sugar and nucleotide sugar metabolic, “ko02020”-ATP-binding cassette (ABC) transporters, “ko00330”-arginine and proline metabolic.^33^

“ko00680” is a methane metabolism pathway that includes methane generation and in methanogenesis metabolism. The proportions of ko00680 in the control group and the experimental groups supplemented with C6-HSL, 3O-C6-HSL, C4-HSL and C8-HSL were 17.78%, 19.85%, 19.63% and 19.09%, respectively. The gene profile of methane growth and metabolism pathway in control group was 16.89%. More “ko00680” were identified in the experimental groups compared with the control group. Fig. 2 also shows that the methanogenic activity of the experimental group is greater than that of the control group. It shows that the experimental group has a stronger ability to metabolize methane. This finding suggests that the addition of signaling molecules to AHLs enhanced gene abundance in the methane metabolic pathway.

In addition, “ko02024” is a quorum-sensing metabolic pathway. In the control group and the experimental group supplemented with C6-HSL, 3O-C6-HSL, C4-HSL and C8-HSL this index accounted for 2.40%, 2.51%, 2.45% and 2.33% respectively, of all pathways. The gene abundance of quorum-sensing metabolic pathway in the control group was 2.14%. Similarly, more genes were identified in the experimental groups compared with the control group. This may be because the addition of signal molecules increases the microbial abundance of anaerobic granular sludge, resulting in increased gene abundance of quorum sensing metabolic pathway.

Methanogens can obtain energy for growth by converting a limited number of substrates to methane under anaerobic conditions. Three types of methanogenic pathways are known: CO_2_ to methane, methanol to methane, and acetate to methane.^34^ By monitoring the unique enzymes in methanogenesis, we can understand the metabolism of methanogens in essence. Fig. 5-b shows the gene abundance of related enzymes in the methane-producing pathway. Coenzyme F420 hydrogenase (EC: 1.12.98.1), formate dehydrogenase (EC: 1.2.1.2) and formyl-furan dehydrogenase (EC: 1.2.99.5) are important enzymes involved in the hydrotrophic methanogenesis pathway.^35^ In the process of methane production, coenzyme F420 hydrogenase acts as an electron carrier during the reaction process, formate dehydrogenase controls the conversion of formic acid to CO_2_ and H_2_, and formyl-furan dehydrogenase catalyzes^36^ the initial step of the conversion of CO_2_ to CH_4_. The relative abundance of important hydrogen-trophic methane-related enzymes involved in the control group was 2.45%, whereas the relative abundances of important hydrogen-trophic methane-related enzymes involved in the experimental group supplemented with C4-HSL, C6-HSL, C8-HSL and O-C6-HSL were 2.78%, 2.50%, 2.78% and 2.78%, respectively. The relative abundance of hydrogen-trophic methane-related enzymes in all the experimental groups was greater than that noted in the control group. These findings indicate that the addition of signal molecules could enhance the methane-producing pathway of hydrogen-trophic AnGS. Acetate kinase (EC: 2.7.2.1), phosphoacetyltransferase (EC: 2.3.1.8), acetyl coenzyme A decarboxylase/synthase complex (ACDs) and acetyl coenzyme A ligase (EC: 6.2.1.1) are key enzymes in the acetic methanogenic pathway. In the control group, the total number of genes involved in the methane-producing pathway of acetic acid was 0.65%, whereas the sums of the genes involved in the methane-producing pathway of acetic acid were 0.73%, 0.68%, 0.73%, and 0.71% for the C4-HSL, C6-HSL, C8-HSL and 3O-C6-HSL treatments, respectively. The gene abundance of the methane-producing pathway in the experimental group was greater than that in the control group, indicating that the methane-producing pathway of acetic acid in the reactor could be enhanced through the addition of signal molecules. The addition of AHLs enhanced both methanogenic pathways, indicating that AHLs can enhance the methanogenic efficiency of AnGS. Microbial diversity analysis showed that the proportion of *Methanobacterium* in the reactor with C6-HSL was less than that in the control group, and the proportion of *Methanosaeta* in the reactor with C4-HSL and 3O-C6-HSL was less than that in the control group. That's probably because the total microbial abundance of anaerobic granular sludge increased after the addition of AHLs, resulting in the reduction of the proportion of methanogenic bacteria in the microbial community. In conclusion, the addition of AHLs can improve the specific methane-producing activity of AnGS by enhancing the methane metabolism pathway of sludge.

## 4. Conclusion

In this experiment, the influence of 4 exogenous AHLS signaling molecules on AnGS was explored. The results showed that C4-HSL, C6-HSL, C8-HSL and 3O-C6-HSL improve the COD removal rate, SMA and organic components of AnGS. In the experimental group, 3O-C6-HSL yielded the best enhancement of methane-producing activity, reaching a maximum of 30.83%. Four different AHLs promote group sensing regulation to stimulate the secretion of EPS in AnGS. C6-HSL and C8-HSL induce the enrichment of *Actinobacteria*, thus accelerating the hydrolysis and acidification of AnGS. C4-HSL, C6-HSL and 3O-C6-HSL enhance the potential methanogenic metabolic pathway of AnGS and further improve the SMA of AnGS. In conclusion, all four AHLs improve the performance of AnGS, potentially providing a novel method for the remediation of damaged sludge and enhancement of the methane production efficiency of the reactor.

## Conflicts of interest

The authors declare that they have no known competing financial interests or personal relationships that could have appeared to influence the work reported in this paper.

## Supplementary Material
